# Circulating endothelial progenitor cells: a new approach to anti-aging medicine?

**DOI:** 10.1186/1479-5876-7-106

**Published:** 2009-12-15

**Authors:** Nina A Mikirova, James A Jackson, Ron Hunninghake, Julian Kenyon, Kyle WH Chan, Cathy A Swindlehurst, Boris Minev, Amit N Patel, Michael P Murphy, Leonard Smith, Doru T Alexandrescu, Thomas E Ichim, Neil H Riordan

**Affiliations:** 1 Bio-Communications Research Institute, Wichita, Kansas, USA; 2 The Center For The Improvement Of Human Functioning International, Wichita, Kansas, USA; 3 The Dove Clinic for Integrated Medicine, Hampshire, UK; 4 Biotheryx Inc, San Diego, California, USA; 5 Novomedix Inc, San Diego, California, USA; 6 Department of Medicine, University of California, San Diego, California, USA; 7 Department of Cardiothoracic Surgery, University of Utah, Salt Lake City, UT, USA; 8 Division of Medicine, Indiana University School of Medicine, IN, USA; 9 Medistem Inc, San Diego, California, USA; 10 Georgetown Dermatology, Washington, DC, USA; 11 Aidan Products, Chandler, Arizona, USA

## Abstract

Endothelial dysfunction is associated with major causes of morbidity and mortality, as well as numerous age-related conditions. The possibility of preserving or even rejuvenating endothelial function offers a potent means of preventing/treating some of the most fearful aspects of aging such as loss of mental, cardiovascular, and sexual function.

Endothelial precursor cells (EPC) provide a continual source of replenishment for damaged or senescent blood vessels. In this review we discuss the biological relevance of circulating EPC in a variety of pathologies in order to build the case that these cells act as an endogenous mechanism of regeneration. Factors controlling EPC mobilization, migration, and function, as well as therapeutic interventions based on mobilization of EPC will be reviewed. We conclude by discussing several clinically-relevant approaches to EPC mobilization and provide preliminary data on a food supplement, Stem-Kine, which enhanced EPC mobilization in human subjects.

## Introduction

The endothelium plays several functions essential for life, including: a) acting as an anticoagulated barrier between the blood stream and interior of the blood vessels; b) allowing for selective transmigration of cells into and out of the blood stream; c) regulating blood flow through controlling smooth muscle contraction/relaxation; and d) participating in tissue remodeling [1]. A key hallmark of the aging process and perhaps one of the causative factors of health decline associated with aging appears to be loss of endothelial function. Whether as a result of oxidative stress, inflammatory stress, or senescence, deficiencies in the ability of the endothelium to respond to physiological cues can alter mental [2], sexual [3], visual [4], and respiratory [5] ability. Specifically, minute alterations in the ability of endothelium to respond to neurotransmitter induced nitric oxide causes profound inability to perform even simple mental functions [6,7]. Small increases in angiogenesis in the retina as a result of injury or glucose are associated with wet macular degeneration blindness [8]. Atherosclerosis of the penile vasculature is a major cause of erectile dysfunction [9]. The pulmonary endothelium's sensitivity to insult can cause hypertension and associated progression to decreased oxygen delivery [10].

Health of the endothelium can be quantified using several methods, including assessment of the physical and mechanical features of the vessel wall, assaying for production of systemic biomarkers released by the endothelium, and quantification of ability of blood vessels to dilate in response to increased flow [11]. Of these, one of the most commonly used assays for endothelium function is the flow mediated dilation (FMD) assay. This procedure usually involves high resolution ultrasound assessment of the diameter of the superficial femoral and brachial arteries in response to reactive hyperemia induced by a cuff. The extent of dilatation response induced by the restoration of flow is compared to dilatation induced by sublingual glyceryl trinitrate. Since the dilatation induced by flow is dependent on the endothelium acting as a mechanotransducer and the dilatation induced by glyceryl trinitrate is based on smooth muscle responses, the difference in dilatation response serves as a means of quantifying one aspect of endothelial health [12,13]. This assay has been used to show endothelial dysfunction in conditions such as healthy aging [14-16], as well as various diverse inflammatory states including renal failure [17], rheumatoid arthritis [18], Crohn's Disease [19], diabetes [20], heart failure [21], and Alzheimer's [22]. Although it is not clear whether reduction in FMD score is causative or an effect of other properties of endothelial dysfunction, it has been associated with: a) increased tendency towards thrombosis, in part by increased von Willibrand Factor (vWF) levels [23], b) abnormal responses to injury, such as neointimal proliferation and subsequent atherosclerosis [24], and c) increased proclivity towards inflammation by basal upregulation of leukocyte adhesion molecules [25].

As part of age and disease associated endothelial dysfunction is the reduced ability of the host to generate new blood vessel [26]. This is believed to be due, at least in part, to reduction of ischemia inducible elements such as the HIF-1 alpha transcription factor which through induction of stromal derived factor (SDF-1) and vascular endothelial growth factor (VEGF) secretion play a critical role in ability of endothelium to migrate and form new capillaries in ischemic tissues [27,28]. Accordingly, if one were to understand the causes of endothelial dysfunction and develop methods of inhibiting these causes or stimulating regeneration of the endothelium, then progression of many diseases, as well as possible increase in healthy longevity may be achieved.

### Endothelial Progenitor Cells: Rejuvenators of the Vasculature

During development endothelial cells are believed to originate from a precursor cell, the hemangioblast, which is capable of giving rise to both hematopoietic and endothelial cells [29]. Classically the endothelium was viewed as a fixed structure with relatively little self renewal, however in the last two decades this concept has fundamentally been altered. The current hypothesis is that the endothelium is constantly undergoing self renewal, especially in response to stress. A key component of endothelial turnover appears to be the existence of circulating endothelial progenitor (EPC) cells that appear to be involved in repair and angiogenesis of ischemic tissues. An early study in 1963 hinted at the existence of such circulating EPC after observations of endothelial-like cells, that were non-thrombogenic and morphologically appeared similar to endothelium, were observed covering a Dacron graft that was tethered to the thoracic artery of a pig [30]. The molecular characterization of the EPC is usually credited to a 1997 paper by Asahara et al. in which human bone marrow derived VEGR-2 positive, CD34 positive monocyte-like cells were described as having ability to differentiate into endothelial cells in vitro and in vivo based on expression of CD31, eNOS, and E-selectin [31]. These studies were expanded into hindlimb ischemia in mouse and rabbit models in which increased circulation of EPC in response to ischemic insult was observed [32]. Furthermore, these studies demonstrated that cytokine-induced augmentation of EPC mobilization elicited a therapeutic angiogenic response. Using irradiated chimeric systems, it was demonstrated that ischemia-mobilized EPC derive from the bone marrow, and that these cells participate both in sprouting of pre-existing blood vessels as well as the initiation of de novo blood vessel production [33]. Subsequent to the initial phenotypic characterization by Asahara et al [31], more detailed descriptions of the human EPC were reported. For example, CD34 cells expressing the markers VEGF-receptor 2, CD133, and CXCR-4 receptor, with migrational ability to VEGF and SDF-1 has been a more refined EPC definition [34]. However there is still some controversy as to the precise phenotype of the EPC, since the term implies only ability to differentiate into endothelium. For example, both CD34+, VEGFR2+, CD133+, as well as CD34+, VEGFR2+, CD133- have been reported to act as EPC [35]. More recent studies suggest that the subpopulation lacking CD133 and CD45 are precursor EPC [36]. Other phenotypes have been ascribed to cells with EPC activity, one study demonstrated monocyte-like cells that expressing CD14, Mac-1 and the dendritic cell marker CD11c have EPC activity based on uptake of acetylated LDL and binding to the ulex-lectin [37,38].

While the initial investigations into the biology of EPC focused around acute ischemia, it appears that in chronic conditions circulating EPC may play a role in endothelial turnover. Apolipoprotein E knockout (ApoE KO) mice are genetically predisposed to development of atherosclerosis due to inability to impaired catabolism of triglyceride-rich lipoproteins. When these mice are lethally irradiated and reconstituted with labeled bone marrow stem cells, it was found that areas of the vasculature with high endothelial turnover, which were the areas of elevated levels of sheer stress, had incorporated the majority of new endothelial cells derived from the bone marrow EPC [39]. The possibility that endogenous bone marrow derived EPC possess such a regenerative function was also tested in a therapeutic setting. Atherosclerosis is believed to initiate from endothelial injury with a proliferative neointimal response that leads to formation of plaques. When bone marrow derived EPC are administered subsequent to wire injury, a substantial reduction in neointima formation was observed [40]. The argument can obviously made that wire injury of an artery does not resemble the physiological conditions associated with plaque development. To address this, Wassmann et al [41], used ApoE KO mice that were fed a high cholesterol diet and observed reduction in endothelial function as assessed by the flow mediated dilation assay. When EPC were administered from wild-type mice restoration of endothelial responsiveness was observed.

In the context of aging, Edelman's group performed a series of interesting experiments in which 3 month old syngeneic cardiac grafts were heterotopically implanted into 18 month old recipients. Loss of graft viability, associated with poor neovascularization, was observed subsequent to transplanting, as well as subsequent to administration of 18 month old bone marrow mononuclear cells. In contrast, when 3 month old bone marrow mononuclear cells were implanted, grafts survived. Antibody depletion experiments demonstrated bone marrow derived platelet derived growth factor (PDGF)-BB was essential in integration of the young heart cells with the old recipient vasculature [42]. These experiments suggest that young EPC or EPC-like cells have ability to integrate and interact with older vasculature. What would be interesting is to determine whether EPC could be "revitalized" ex vivo by culture conditions or transfection with therapeutic genes such as PDGF-BB.

Given animal studies suggest EPC are capable of replenishing the vasculature, and defined markers of human EPC exist, it may be possible to contemplate EPC-based therapies. Two overarching therapeutic approaches would involve utilization of exogenous EPC or mobilization of endogenous cells. Before discussing potential therapeutic interventions, we will first examine several clinical conditions in which increasing circulating EPC may play a role in response to injury.

### Clinical Increase of Circulating EPC as a Response to Injury

Tissue injury and hypoxia are known to generate chemoattractants that potentially are responsible for mobilization of EPC. Reduction in oxygen tension occurs as a result of numerous injuries including stroke, infarction, or contusion. Oxygen tension is biologically detected by the transcription factor HIF-1 alpha, which upon derepression undergoes nuclear translocation. This event causes upregulated expression of a plethora of angiogenesis promoting cytokines and chemoattractants [43], such as stromal derived factor (SDF)-1 and VEGF [44,45]. On the other hand, tissue necrosis causes release of "danger signals" such as HMBG1, a nuclear factor that has direct chemoattractant activity on mesoangioblasts, a type of EPC [46,47]. It has been demonstrated that this systemic release of chemoattractant cytokines after vascular injury or infarct is associated with mobilization of endogenous bone marrow cells and EPC [48].

Myocardial infarction has been widely studied in the area of regenerative medicine in which cellular and molecular aspects of host response post-injury are relatively well defined. EPC mobilization after acute ischemia has been demonstrated in several cardiac infarct studies. This was first reported by Shintani et al who observed increased numbers of CD34 positive cells in 16 post infarct patients on day 7 as compared to controls. The rise in CD34 cells correlated with ability to differentiate into cells morphologically resembling endothelium and expressing endothelial markers KDR and CD31. Supporting the concept that response to injury stimulates EPC mobilization, a rise in systemic VEGF levels was correlated with increased EPC numbers [45]. A subsequent study demonstrated a similar rise in circulating EPC post infarct. Blood was drawn from 56 patients having a recent infarct (<12 hours), 39 patients with stable angina, and 20 healthy controls. Elevated levels of cells expressing CD34/CXCR4+ and CD34/CD117+ and c-met+ were observed only in the infarct patients which were highest at the first blood draw. In this study the mobilized cells not only expressed endothelial markers, but also myocytic and cardiac genes [49]. The increase in circulating EPC at early timepoints post infarction has been observed by other groups, and correlated with elevations in systemic VEGF and SDF-1 [50,51].

In the case of cerebral infarction studies support the concept that not only are EPC mobilized in response to ischemia, but also that the extent of mobilization may be associated with recovery. In a trial of 48 patients suffering primary ischemic stroke, mobilization of EPC was observed in the first week in comparison to control patients. EPC were defined as cells capable of producing endothelial colony forming units. A correlation between improved outcome at 3 months and extend of EPC mobilization was observed based on the NIHSS and Rankin score [52]. In a similar study, Dunac et al reported on circulating CD34 levels of 25 patients with acute stroke for 14 days. A correlation between improvement on the Rankin scale and increased circulating CD34 cells was reported [53]. Noteworthy was that the level of CD34 mobilization was similar to that observed in patients treated with the mobilize G-CSF. In a larger study, Yip et al examined EPC levels in 138 consecutive patients with acute stroke and compared them to 20 healthy volunteers and in 40 at-risk control subjects [54]. Three EPC phenotypes were assessed by flow cytometry at 48 hours after stroke: a) CD31/CD34, b) CD62E/CD34, and c) KDR/CD34. Diminished levels of all three EPC subsets in circulation was predictive of severe neurological impairment NIHSS >/= 12, while suppressed levels of circulating CD31/34 cells was correlated with combined major adverse clinical outcomes as defined by recurrent stroke, any cause of death, or NIHSS >/= 12. Increased levels of the KDR/CD34 phenotype cells was strongly associated with NIHSS > or = 4 on day 21. Although these studies do not directly demonstrate a therapeutic effect of the mobilized EPC, animal studies in the middle cerebral artery ligation stroke model have demonstrated positive effects subsequent to EPC administration [55,56], an effect which appears to be at least partially dependent on VEGF production from the EPC [57].

Another ischemia-associated tissue insult is acute respiratory distress syndrome (ARDS), in which respiratory failure often occurs as a result of disruption of the alveolar-capillary membrane, which causes accumulation of proteinaceous pulmonary edema fluid and lack of oxygen uptake ability [58]. In this condition there has been some speculation that circulating EPC may be capable of restoring injured lung endothelium. For example, it is known that significant chimerism (37-42%) of pulmonary endothelial cells occurs in female recipients of male bone marrow transplants [59]. Furthermore, in patients with pneumonia infection there is a correlation between infection and circulating EPC, with higher numbers of EPC being indicative of reduced fibrosis [60]. The possibility that EPC are mobilized during ARDS and may be associated with benefit was examined in a study of 45 patients with acute lung injury in which a correlation between patients having higher number of cells capable of forming endothelial colonies in vitro and survival was made. Specifically, the patients with a colony count of >or= 35 had a mortality of approximately 30%, compared to patients with less than 35 colonies, which had a mortality of 61%. The correlation was significant after multivariable analysis correcting for age, sex, and severity of illness [61]. From an interventional perspective, transplantation of EPC into a rabbit model of acute lung injury resulted in reduction of leukocytic infiltrates and preservation of pulmonary cellular integrity [62].

Sepsis is a major cause of ARDS and is associated with acute systemic inflammation and vascular damage. Septic patients have elevated levels of injury associated signals and EPC mobilizers such as HMGB1 [63], SDF-1 [64], and VEGF [65]. Significant pathology of sepsis is associated with vascular leak and disseminated intravascular coagulation [66]. The importance of the vasculature in sepsis can perhaps be supported by the finding that the only drug to have an impact on survival, Activated Protein C, acts primarily through endothelial protection [67]. Septic patients are known to have increased circulating EPC as compared to controls. Becchi et al observed a correlation between VEGF and SDF-1 levels with a 4-fold rise in circulating EPC in septic patients as compared to healthy controls [64]. A correlation between EPC levels and survival after sepsis was reported in a study of 32 septic patients, 15 ICU patients, and 15 controls. Of the 8 patients who succumbed to sepsis by 28 days, as compared to 24 survivors, a significantly reduced EPC number in non-survivors was reported [68].

It appears that in conditions of acute injury, elevation of EPC in circulation occurs. Although studies in stroke [52-54], ARDS [61], and sepsis [68] seem to correlate outcome with extend of mobilization, work remains to be performed in assessing whether it is the EPC component that is responsible for benefits or other confounding variables. Taking into account the possibility that EPC may act as an endogenous repair mechanism, we will discuss data in chronic degenerative conditions in which circulating EPC appear to be suppressed.

### Chronic Inflammatory Disease Inhibit Circulating EPC

There is need for angiogenesis and tissue remodeling in the context of various chronic inflammatory conditions. However in many situations it is the aberrant reparative processes that actually contribute to the pathology of disease. Examples of this include: the process of neointimal hyperplasia and subsequent plaque formation in response to injury to the vascular wall [69], the process of hepatic fibrosis as opposed to functional regeneration [70], or the post-infarct pathological remodeling of the myocardium which results in progressive heart failure [71]. In all of these situations it appears that not only the lack of regenerative cells, but also the lack of EPC is present. Conceptually, the need for reparative cells to heal the ongoing damage may have been so overwhelming that it leads to exhaustion of EPC numbers and eventual reduction in protective effect. Supporting this concept are observations of lower number of circulating EPC in inflammatory diseases, which may be the result of exhaustion. Additionally, the reduced telomeric length of EPC in patients with coronary artery disease [72], as well as reduction of telomere length in the EPC precursors that are found in the bone marrow [73,74] suggests that exhaustion in response to long-term demand may be occurring. If the reparatory demands of the injury indeed lead to depletion of EPC progenitors, then administration of progenitors should have therapeutic effects.

Several experiments have shown that administration of EPC have beneficial effects in the disease process. For example, EPC administration has been shown to: decrease balloon injury induced neointimal hyperplasia [75], b) suppress carbon tetrachloride induced hepatic fibrosis [76,77], and inhibit post cardiac infarct remodeling [78]. One caveat of these studies is that definition of EPC was variable, or in some cases a confounding effect of coadministered cells with regenerative potential may be present. However, overall, there does appear to be an indication that EPC play a beneficial role in supporting tissue regeneration. As discussed below, many degenerative conditions, including healthy aging, are associated with a low-grade inflammation. There appears to be a causative link between this inflammation and reduction in EPC function.

Inflammatory conditions present with features, which although not the rule, appear to have commonalities. For example, increases in inflammatory markers such as C-reactive protein (CRP), erythrocyte sedimentation rate, and cytokines such as TNF-alpha and IL-18 have been described in diverse conditions ranging from organ degenerative conditions such as heart failure [79,80], kidney failure [81,82], and liver failure [83,84] to autoimmune conditions such as rheumatoid arthritis [85] and Crohn's Disease [86], to healthy aging [87,88]. Other markers of inflammation include products of immune cells such as neopterin, a metabolite that increases systemically with healthy aging [89], and its concentration positively correlates with cognitive deterioration in various age-related conditions such as Alzheimer's [90]. Neopterin is largely secreted by macrophages, which also produce inflammatory mediators such as TNF-alpha, IL-1, and IL-6, all of which are associated with chronic inflammation of aging [91]. Interestingly, these cytokines are known to upregulate CRP, which also is associated with aging [92]. While there is no direct evidence that inflammatory markers actively cause shorted lifespan in humans, strong indirect evidence of their detrimental activities exists. For example, direct injection of recombinant CRP in healthy volunteers induces atherothrombotic endothelial changes, similar to those observed in aging [93]. In vitro administration of CRP to endothelial cells decreases responsiveness to vasoactive factors, resembling the human age-associated condition of endothelial hyporesponsiveness [94].

Another important inflammatory mediator found elevated in numerous degenerative conditions is the cytokine TNF-alpha. Made by numerous cells, but primarily macrophages, TNF-alpha is known to inhibit proliferation of repair cells in the body, such as oligodendrocytes in the brain [95], and suppress activity of endogenous stem cell pools [96,97]. TNF-alpha decreases EPC viability, an effect that can be overcome, at least in part by antioxidant treatment [98]. Administration of TNF-alpha blocking agents has been demonstrated to restore both circulating EPC, as well as endothelial function in patients with inflammatory diseases such as rheumatoid arthritis [18,99,100],

It appears that numerous degenerative conditions are associated with production of inflammatory mediators, which directly suppress EPC production or activity. This may be one of the reasons for findings of reduced EPC and FMD indices in patients with diverse inflammatory conditions. In addition to the direct effects, the increased demand for de novo EPC production in inflammatory conditions would theoretically lead to exhaustion of EPC precursors cells by virtue of telomere shortening.

### EPC Exhaustion as a Mechanism of Chronic Inflammation

On average somatic cells can divide approximately 50 times, after which they undergo senescence, die or become cancerous. This limited proliferative ability is dependent on the telomere shortening problem. Every time cells divide the ends of the chromosomes called "telomeres" (complexes of tandem TTAGGGG repeats of DNA and proteins), are not completely replicated, thus they progressively get shorter [101]. Once telomeres reach a critical limit p53, p21, and p16 pathways are activated as a DNA damage response reaction instructing the cell to exit cell cycling. Associated with the process of senescence, the cells start expressing inflammatory cytokines such as IL-1 [102,103], upregulation of adhesion molecules that attract inflammatory cells such as monocytes [104,105], and morphologically take a flattened, elongated appearance. Physiologically, the process of cellular senescence caused in response to telomere shortening is believed to be a type of protective mechanism that cells have to prevented carcinogenesis [106]. At a whole organism level the association between telomere length and age has been made [107], as well, disorders of premature aging such as ataxia telangiectasia are characterized by accelerated telomere shortening [108].

The importance of this limited proliferative ability becomes apparent in our discussion of EPC. In general there is a need for continual endothelial cell replacement from EPC. Because the endothelial cells are exposed to enormous continual sheer stress of blood flow, mechanisms of repair and proliferation after injury need to exist. Theoretically, the more sheer stress on a particular artery, the more cell division would be required to compensate for cell loss. Indeed this appears to be the case. For example, telomeres are shorter in arteries associated with higher blood flow and sheer stress (like the iliac artery) as compared to arteries of lower stress such as the mammary artery [109]. The theory that senescence may be associated with atherosclerosis is supported since the iliac artery, which is associated with higher proliferation of endothelial cells and is also at a higher risk of atherosclerosis, thus prompting some investigators to propose atherosclerosis being associated with endothelial senescence [110,111].

In an interesting intervention study Satoh et al examined 100 patients with coronary artery disease and 25 control patients. Telomere lengths were reduced in EPC of coronary artery disease patients as compared to controls. Lipid lowering therapy using agents such as atorvastatin has previously been shown to reduced oxidative stress and increase circulating EPC. Therapy with lipid lowering agents in this study resulted in preservation of telomeric length, presumably by decreasing the amount of de novo EPC produced, as well as oxidative stress leading to telomere erosion [112]. One important consideration when discussing telomere shortening of EPC is the difference between replicative senescence, which results from high need for differentiated endothelial cells, and stress induced senescence, in which inflammatory mediators can directly lead to telomere shortening. For example, smoking associated oxidative stress has been linked to stress induced senescence in clinical studies [113], whereas other studies have implicated inflammatory agents such as interferon gamma [114], TNF-alpha [115], and oxidative mediators as inducers of stress induced senescence [116].

### Intervening to Increase Vascular Health and EPC

Based on the above descriptions, it appears that in degenerative conditions, as well as in aging, an underlying inflammatory response occurs that is directly or indirectly associated with inhibition of circulating EPC activity. Directly, inflammation is known to suppress stem cell turnover and activity of EPC. Indirectly, inflammatory conditions place increased demands on the EPC progenitors due to overall increased need for EPC. Accordingly, an intervention strategy may be reduction in inflammatory states: this may be performed in a potent means by administration of agents such as TNF blockers [55], or more chronically by dietary supplements [117,118], caloric restriction [119], exercise [120,121], consuming blueberries [122], green tea [123], or statin therapy [124]. One example of a large scale intervention was the JUPITER trial of >17,000 healthy persons without hyperlipidemia but with elevated high-sensitivity C-reactive protein levels, Crestor significantly reduced the incidence of major cardiovascular incidents as well as lowering CRP levels [124]. Crestor has been shown to increase circulating EPC levels in vivo [125], in part through reduction of detrimental effects of asymmetric dimethylarginine on EPC [126].

Besides attempting to reduce inflammation, administration of EPC is another therapeutic possibility. The area of cardiac regeneration has been subject to most stem cell investigation besides hematopoietic reconstitution. Specifically, several double blind studies have been performed demonstrating overall increased cardiac function and reduction in pathological remodeling subsequent to administration of autologous bone marrow mononuclear cells [127-129]. Original thoughts regarding the use of bone marrow stem cells in infarcts revolved around studies showing "transdifferentiation" of various bone marrow derived cells into cells with myocardial features [130,131]. While this concept is attractive, it has become very controversial in light of several studies demonstrating extremely minute levels of donor-derived cardiomyocytes, despite clinical improvement [132,133]. An idea that has attracted interest is that bone marrow cells contain high numbers of EPC [134], so the therapeutic effect post infarct may not necessarily need to be solely based on regeneration via transdifferentiation, but via production of new blood vessels in the injured myocardium mediated by administered EPC in the bone marrow [135]. This view is supported by studies demonstrating that administration of EPC in other conditions of injury or fibrotic healing results in reduced tissue damage and organ functionality.

Instead of administering EPC another therapeutic possibility is to "reposition" them or simply to mobilize them from bone marrow sources. As previously discussed, myocardial and cerebral infarcts seem to cause a "natural mobilization", which may be part of the endogenous response to injury. These observations led investigators to assess whether agents that mobilize EPC may be used therapeutically. Granulocyte colony stimulating factor (G-CSF) has been used clinically for mobilization of hematopoietic stem cells (HSC) for more than a decade during donor stem cell harvesting. Mechanistically G-CSF is believed to induce a MMP-dependent alteration of the SDF-1 gradient in the bone marrow [136,137], as well as function through a complement-dependent remodeling of the bone marrow extracellular matrix [138,139]. It was found that in addition to mobilizing HSC, G-CSF stimulates mobilization of EPC as well, through mechanisms that are believed to be related [35,140]. Several studies have been performed in which G-CSF was administered subsequent to infarct. Although it is impossible to state whether the mobilization of HSC or EPC accounted for the beneficial effects, we will overview some of these studies.

The Front-Integrated Revascularization and Stem Cell Liberation in Evolving Acute Myocardial Infarction by Granulocyte Colony-Stimulating Factor (FIRSTLINE-AMI) trial evaluated 30 patients with ST-elevation myocardial infarction treated with control or G-CSF after successful revascularization [141]. Fifteen patients received 6 days of G-CSF at 10 μg/kg body weight, whereas the other 15 received standard care only. Four months after the infarct, the group that received G-CSF possessed a thicker myocardial wall at the area of infarct, as compared to controls. This was sustained over a year. Statistically significant improvements in ejection fraction, as well as inhibition of pathological remodeling was observed in comparison to controls. A larger subsequent study with 114 patients, 56 treated and 58 control demonstrated "no influence on infarct size, left ventricular function, or coronary restenosis" [142]. There may be a variety of reasons to explain the discrepancy between the trials. One most obvious one is that the mobilization was conducted immediately after the heart attack, whereas it may be more beneficial to time the mobilization with the timing of the chemotactic gradient released by the injured myocardium. This has been used to explain discrepancies between similar regenerative medicine trials [143]. Supporting this possibility is a study in which altered dosing was used for the successful improvement in angina [144]. Furthermore, a recent study last year demonstrated that in 41 patients with large anterior wall AMI an improvement in LVEF and diminished pathological remodeling was observed [145]. Thus while more studies are needed for definitive conclusions, it appears that there is an indication that post-infarct mobilization may have a therapeutic role. In the future, other clinically-applicable mobilizers may be evaluated. For example, growth hormone, which is used in "antiaging medicine" has been demonstrated to improve endothelial responsiveness in healthy volunteers [146], and patients with congestive heart failure [147], this appears to be mediated through mobilization of endothelial progenitor cells [148,149].

## Conclusions: Nutraceutical Based Mobilization of EPC

One area of recent interest in the biomedical field has been functional foods and nutraceuticals. While it is known that alteration of diet may modulate FMD responses, to our knowledge, little work as been reported on dietary-supplements altering levels of circulating EPC. The nutritional supplement Stem-Kine (Aidan Products, Chandler, AZ) contains: ellagic acid a polyphenol antioxidant found in numerous vegetables and fruits; vitamin D3 which has been shown to mildly increase circulating progenitor cells; beta 1,3 glucan (previous studies have reported administration of various beta glucans to elicit stem cell mobilization [150]), and a ferment of the bacterium, *Lactobacillus fermentum*. *Lactobacillus fermentum *is generally regarded as safe, and has been in the food supply for hundreds of years as a starter culture for the production of sour dough bread and provides for its characteristic sour flavor. Extract of green tea, extract of goji berries, and extract of the root of astragalus were added prior to the fermentation process. Green tea extracts and some components of goji berries are known to mildly stimulate progenitor cell release, and astragalosides and other molecules found in the root of astragalus are known antioxidants that can prevent cellular damage secondary to oxidation. Fermentation is known to increase the bioavailability of minerals, proteins, peptides, antioxidants, flavanols and other organic molecules. Imm-Kine, another *Lactobacillus fermentum *fermented product that includes beta 1,3, glucan has been safely distributed for 9 years without reported side effects.

We report here data from 6 healthy volunteers supplemented with StemKine (under an approved IRB protocol) for a period of 14 days (two capsules, am, two capsules pm, by mouth--700 mg per capsule). To our knowledge this is the first report of a combination of naturally occurring molecules from food products altering the levels of circulating EPCs in humans.

**Figure 1:**
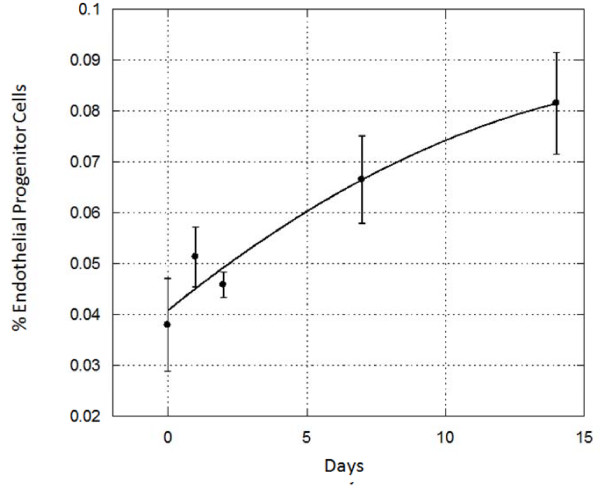
**Stem-Kine Supplementation Augments Circulating EPC**. StemKine was administered at a concentration of 2,800 mg/day to 6 healthy volunteers. Flow cytometric analysis of cells double-staining for VEGFR2 and CD34 was performed with samples extracted at the indicated timepoints. Y-axis represents percentage double positive cells from cells.

As seen in Figure 1, an increase in cells expressing VEGFR2 and CD34 was observed, which was maintained for at least 14 days. These data suggest the feasibility of modulating circulating EPC levels using food supplements. Future studies integrating natural products together with regenerative medicine concepts may lead to formulation of novel treatment protocols applicable to age-associated degeneration.

## Competing interests

NHR is a shareholder of Aidan Products. All other authors have no competing interests.

## Authors' contributions

NHR and NAM designed experiments, interpreted data and conceptualized manuscript. RH, JK, KWA, CAS, BM, ANP, MPM, LS, DTA, and TEI provided detailed ideas and discussions, and/or writing of the manuscript. NAM and JAJ performed the experiments. All authors read and approved the final manuscript.
